# International Epidemic Intelligence at the Institut de Veille Sanitaire, France

**DOI:** 10.3201/eid1310.070522

**Published:** 2007-10

**Authors:** Brice Rotureau, Philippe Barboza, Arnaud Tarantola, Christophe Paquet

**Affiliations:** * Institut de Veille Sanitaire, Saint-Maurice, France

**Keywords:** Epidemic intelligence, international health surveillance, information, dispatch

## Abstract

The French Institute for Public Health Surveillance monitors health events of potential international importance occurring worldwide to provide timely warning to French health authorities. We reviewed the nature and place of occurrence of the last 200 events. From an individual country’s perspective, the need for multiple sources is emphasized.

Local epidemics may rapidly acquire international importance due to international travel and trade (*1*). With early warning, timely and adequate control measures can be adopted to prevent transmission. Epidemic intelligence is the systematic collection and collation of verified and unverified information from various sources, such as governments, United Nations organizations, nongovernmental organizations (NGOs), mass media, and personal communications (*2*). The Internet is transforming global disease surveillance (*3*). Information is selected by specific criteria, verified (if informal), and thoroughly analyzed before communication to the public.

The Institut de Veille Sanitaire (InVS)—the French Institute for Public Health Surveillance—set up an epidemic intelligence unit to monitor outbreaks worldwide along the lines of the World Health Organization (WHO) Department of Epidemic and Pandemic Alert and Response. At the national level, this unit’s legal mandate is to detect, verify, and rapidly assess information on potential international health threats, which may affect populations in France or French nationals worldwide. Its main task is to inform French authorities, public health professionals, and other partners of these epidemic risks and to place events with excessive media coverage in the proper perspective. Information is structured and widely communicated weekly through the electronic Bulletin Hebdomadaire International (BHI) (www.invs.sante.fr/international/index.htm). To better assess the type, characteristics, and location of alerts documented by the unit, we reviewed the health events posted in the BHI, i.e., all confirmed information on potential international health threats that may affect populations in France or French nationals worldwide. We also examined initial signals and the use of various sources of international epidemic intelligence.

## The Study

We reviewed 200 events posted in the 32 BHI from May 17, 2006 to December 27, 2006. We examined event topics, geographic location (country and world region), onset date of the first case/outbreak, source, and publication date of the first signal, delay between first occurrence and the first signal, signal type (passive email alert vs. active manual search) and alarm status (first report vs. follow-up).

Potential sources were formal outbreak reports communicated by countries and supranational organizations (both official and NGO) posted on the Internet and scientific online forums such as ProMED-mail (available from www.promedmail.org) (*4**–**6*). A dedicated tool was used to collect information available on the Internet: the Global Public Health Intelligence Network (GPHIN) (available from www.phac-aspc.gc.ca/media/nr-rp/2004/2004_gphin-rmispbk_e.html) is a software codeveloped by WHO and Health Canada. GPHIN is a secure, restricted-access early warning system that gathers media reports of public health significance on a 24/7 basis (*7*). Like the medical intelligence system developed by the European community, the GPHIN is a multilingual system that provides relevant unverified information on public health events by monitoring global media sources in 7 languages. This automated process includes a filter for relevancy, but specific email alerts and the categorizing of information must be complemented by human analysis.

The highest proportion of events (53%, 105/199) occurred in Asia (Table 1). Of these events, 61% (122/199) were highly pathogenic avian influenza (HPAI) (H5N1) infections in animals or humans. These, combined with multicountry outbreaks of cholera (8%), chikungunya virus disease (7%), dengue (7%), and poliomyelitis (6%), were the most recurring topics posted in the BHI (Table 2).

**Table 1:** Events posted in the BHI from May 17 through December 27, 2006, by world region*

Region	No. events		
	HPAI (H5N1)	Other	Total
Africa	21	29	50
Americas	0	11	11
Asia	80	25	105
Europe	20	9	29
Middle East	1	3	4
Total	122	77	199

*BHI, Bulletin Hebdomadaire International; HPAI, highly pathogenic avian influenza.

**Table 2:** Events posted in the BHI from May 17 through December 27, 2006, by topic*

Event	No. (%)
HPAI (H5N1) in animals	67 (34)
HPAI (H5N1) in human	55 (28)
Cholera	16 (8)
Chikungunya	13 (7)
Dengue fever	13 (7)
Poliomyelitis	11 (6)
Malaria	6 (3)
Japanese encephalitis	4 (2)
Adulterated alcohol intoxication	3 (2)
Crimean-Congo hemorragic fever	3 (2)
Plague	2 (1)
Yellow fever	2 (1)
Deaths following influenza vaccination	1 (0.5)
Measles	1 (0.5)
Micro-algae intoxication	1 (0.5)
Rift Valley fever	1 (0.5)
Viral meningitis	1 (0.5)
Total	200 (100)

*BHI, Bulletin Hebdomadaire International; HPAI, highly pathogenic avian influenza.

The first signal’s source could be identified in 88% (176/200) of events. News reports collected using the GPHIN were the most important initial sources of information, providing 36% (63/176) of all initial signals (Appendix Table). Of these 63 events, 37 (59%) were automatically forwarded by the GPHIN e-alert system, and 26 (41%) were detected through active searches. Official signals from the WHO network accounted for 29% (51/176) of all events posted in the BHI; ProMED-mail provided the first signal for 17% of the events. Of 176 events included in the BHI, 20% (35/176) were first detected only by manual and nonspecific Internet searches. Furthermore, 60% (105/176) of the posted events were first detected through informal sources that required extensive verification.

On average, delay between the first case of an outbreak (including retrospectively) and the first widely available signal was 3 months and 14 days (range 1 day–2 years, 10.5 months; n = 86). Among the 200 events posted, 107 were reported for the first time. For these, mean delay between the first case and the first signal was 1 month and 23 days (range 1 day–5 months, 16 days; n = 63). Based on a small sample of alerts, the mean delay for alert messages provided by the GPHIN was shorter (1 month, 19 days; n = 19) than that of the ProMED-mail (2 months, 4 days; n = 6).

## Conclusions

As in the 2006 WHO report (*2*), influenza A (H5N1) HPAI cases and cholera outbreaks were the major topics included in the BHI. Alerts posted for this period mainly concerned Asia due to the occurrence of influenza A (H5N1). Due to the specific economic and political situations of each country, availability and sensitivity of information sources differed. Multiple information sources somewhat compensated for these differences. Delays between the occurrence of events and first reports reflect the following: 1) interval between the occurrence of a first case and development into a full-blown outbreak of international importance; 2) limitations of communicable disease surveillance, i.e., interval before an event is detected; 3) intrinsic limitations of epidemic intelligence, i.e., availability of information; and 4) the unavoidable prioritization of alerts, given time and resource constraints.

GPHIN was more efficient than any other information source used in this analysis, including ProMED-mail, both in terms of number of signals and rapidity of signal availability after event occurrence. Signals from GPHIN, however, are unverified media reports,and not all relevant events for our specific needs are electronically forwarded as e-alert. Permanent proactive searches using GPHIN or similar tools remain compulsory to address this limitation. Time-consuming human-operated screening of each report of the daily GPHIN list (500–2,000 reports/day) is needed as signal detection cannot be automated. Verification processes are essential because reports of outbreaks are widely disseminated and easily accessible to the public (*1*).

WHO and other supranational organizations, such as the European Centre for Disease Prevention and Control, monitor health events of international importance. However, these organizations cannot completely meet all needs of individual countries. Our experience at a national institute shows that implementation of epidemic intelligence should be specifically tailored to effectively monitor the health of a country’s population and translate directly into public health action. For example, in 2005, an extensive cholera outbreak was detected and documented in Senegal, with far-reaching implications for Franco-Senegalese pilgrims. Information posted in the BHI is used by physicians in French tropical disease departments and travel clinics, who can provide timely information to travelers, and target clinical examinations of those returning with suggestive symptoms. The operational suspect case definition for influenza A (H5N1) in returning travelers is continuously updated as foci appear in various areas. Information is also forwarded to the French Ministries of Health and Foreign Affairs to alert a larger segment of the population through institutional websites or warnings on airport billboards.

## Supplementary Material

Appendix Table Events posted in the BHI from May 17, through December 27, 2006, by source of the first signal*
