# Supplementary material for: International Epidemic Intelligence at the Institut de Veille Sanitaire, France
Source: Emerg Infect Dis. 2007 Oct;13(10):1590–2. doi: 10.3201/eid1310.070522 (PMC2851537; doi:10.3201/eid1310.070522)
Supplement: Appendix Table — Events posted in the BHI from May 17, through December 27, 2006, by source of the first signal* [file 07-0522_appT-s1.pdf]

Appendix Table. Events posted in the BHI from May 17, through December 27, 2006, by source of the first signal*

Source of the first signal	No. events (%)
GPHIN	63 (36)
World Health Organization network	51 (29)
ProMED-mail	30 (17)
Agence France Presse	11 (6)
Office International des Epizooties	9 (5)
Ministries	7 (4)
French Health Directorate	2 (1)
Regional surveillance networks	1 (0.6)
Scientific peer-reviewed literature	1 (0.6)
Electronic discussion group	1 (0.6)
Total	176 (100)

*BHI, Bulletin Hebdomadaire International; GPHIN, Global Public Health Intelligence Network.
